# The Anticoccidial In Vitro Effects and Antioxidant Properties of Several Plants Traditionally Used for Coccidiosis in Togo

**DOI:** 10.3390/vetsci11080345

**Published:** 2024-07-31

**Authors:** Ferdinand Grégoire Tchodo, Hervé Brice Dakpogan, Sabrina Sanvee, Benjamin Adjei-Mensah, Claude Cocou Kpomasse, Simplice Karou, Wéré Pitala, Kokou Tona, Batomayena Bakoma

**Affiliations:** 1 Regional Center of Excellence in Poultry Science, University of Lome, Lome BSP 1515, Togo; 2 School of Animal Production System Management, National Agricultural University of Ketou, Ketou P.O. Box 43, Benin; 3 Pharmaceutical Sciences Research Laboratory, University of Lome, Lome BSP 1515, Togo

**Keywords:** plant extracts, anticoccidial activities, antioxidant, chicken oocysts, in vitro

## Abstract

**Simple Summary:**

Medicinal plants rich in biologically active phytochemicals have been extensively utilized to treat diseases affecting livestock. This paper demonstrates the efficacy of herbal remedies, including *Carica papaya* seeds, *Combretum micranthum* leaves, *Sarcocephalus latifolius* roots, *Azadirachta indica* leaves, and *Vernonia amygdalina* leaves, traditionally used in poultry farms in Togo, in inhibiting *Eimeria* spp. sporulation in vitro. The most successful extracts were found to be those from *Sarcocephalus latifolius* roots, *Azadirachta indica* leaves, and *Carica papaya* seeds, which at 75 mg/mL were able to block up to 75% of oocyst sporulation. The maximum antioxidant activity was, likewise, found in the root extract of *Sarcocephalus latifolius*. The presence of significant medicinal phytochemicals like flavonoids and phenols in the plant extracts was thought to be responsible for these advantageous qualities. This study suggests that these plants could be used to develop new treatments or an anticoccidial complex against coccidiosis, an important disease affecting poultry.

**Abstract:**

Coccidiosis is a parasitic disease that often affects livestock. Identifying plants with inhibitory effects on the development of the parasite could help in finding new natural treatments. This study aimed to evaluate the anticoccidial potentials of extracts from *Azadirachta indica* leaves (AILs), *Combretum micranthum* leaves (CMLs), *Carica papaya* seeds (CPSs), *Sarcocephalus latifolius* roots (SLRs), and *Vernonia amygdalina* leaves (VALs). The in vitro anticoccidial efficacy of the extracts was evaluated through oocyst sporulation inhibition and sporozoite viability inhibition assays of *Eimeria* oocysts. The setup was examined for 72 h (every 24 h) of incubation. The DPPH radical scavenging activity and ferric reducing antioxidant power were used to evaluate the antioxidant potential of the extracts. Among the tested extracts, the SLR, CPS, and AIL extracts exhibited the maximum oocyst sporulation inhibition (75.85 ± 1.21%, 74.53 ± 1.65%, and 71.58 ± 0.24%, respectively) at a concentration of 75 mg/mL of plant extracts against the *Eimeria* species. The *Sarcocephalus latifolius* root extract showed the highest radical scavenging capacity (76.25 ± 0.53) and reducing power (86.21 ± 4.28). The biochemical screening of the selected plant extracts revealed the presence of antioxidant compounds such as phenols, flavonoids, alkaloids, saponins, and carbohydrates. The SLR extract contained the highest amounts of phenols (56.11 ± 0.33 µg/mL) and flavonoids (36.65 ± 1.85 µg/mL). In conclusion, the selected hydro-ethanolic extracts from these plants possess excellent anticoccidial and antioxidant activities, which can be attributed to the presence of medicinally important phytochemicals. Further research is needed to identify and isolate the active anticoccidial compounds from these plants, which could be utilized in the development of drugs against coccidiosis.

## 1. Introduction

Coccidiosis is a significant poultry disease caused by single-celled protozoa of the genus Eimeria, including *Eimeria acervulina*, *Eimeria brunetti*, *Eimeria maxima*, *Eimeria mitis*, *Eimeria necatrix*, *Eimeria praecox*, *Eimeria tenella*, *Eimeria lata*, *Eimeria nagambie*, and *Eimeria zaria*, which are known to be the species responsible for coccidiosis in chickens (*Gallus gallus domesticus*) [1,2]. Among them, *E. tenella* and *E. necatrix* are recognized to be the most pathogenic, followed by *E. maxima* and *E. Brunetti* [2]. The deadly coccidiosis condition affects the gastrointestinal tract [3] and induces a severe inflammatory response, leading to tissue oxidative stress, lipid peroxidation damage, diarrheal bleeding, a poor feed conversion ratio, decreased productivity, increased susceptibility to other diseases, and even fatality [4]. It is estimated that annual global losses resulting from Eimeria infection surpass GBP 10.36 billion [5]. The life cycle of this parasite is intricate and starts when sporulated oocysts from the litter are inadvertently consumed. It then progresses to the daily excreta of millions of non-sporulated oocysts from infected hens. Sporocysts are released in the host organism once the sporulated oocysts enter the gizzard due to mechanical friction. Under the action of bile and trypsin, sporozoites break free from the sporocysts and infiltrate the intestinal mucosal epithelial cells, where they undergo schizogony and gametogony [6]. Since the invasion of epithelial cells by sporozoites is a prerequisite for the development of coccidiosis, this invasion serves as a primary target for anticoccidial medications [7]. Consequently, one of the most important aspects of controlling this protozoan is to disrupt the sporulation process.

Anticoccidial medications have been a common approach for a long time to manage avian coccidiosis in contemporary chicken farming. Despite the effectiveness and affordability of this approach, the rise in drug resistance and consumer demands for meat devoid of residues, among other reasons, has prompted the creation of substitute control methods [8]. There is, therefore, the need for alternative treatments of poultry diseases including coccidiosis. Using plant extracts as medications could help with these issues because they are not just natural items but might also contain novel therapeutic compounds against which resistance has not yet been acquired. In this context, herbal anticoccidial can serve as an alternative to conventional treatments, particularly in developing countries [9]. Several investigations have shown that plant extracts contain naturally occurring physiologically active substances such as flavonoids and tannins, which can serve as preventive anticoccidial agents and stimulate the host immune system to shield the intestinal liners against bacterial invasion [1,10]. In Togo’s traditional poultry system, *Azadirachta indica* leaves (AILs), *Combretum micranthum* leaves (CMLs), *Carica papaya* seeds (CPSs), *Sarcocephalus latifolius* roots (SLRs), and *Vernonia amygdalina* leaves (VALs) are the plants that are most used as antiparasitic herbal extracts [11]. It was hypothesized that these herbal extracts could reduce the number of viable sporozoites in the growth medium. Therefore, the purpose of this current study was to assess the effects of these particular plant extracts’ phytochemical components, antioxidant activities, and in vitro anticoccidial potentials on the sporulation and survivability of oocysts isolated from chickens that belong to the *Eimeria* spp. 

## 2. Materials and Methods

### 2.1. Chemicals and Reagents

The reagents for the preparation of the Phosphate Buffered Saline (PBS), 2.5% potassium dichromate (K_2_Cr_2_O_7_), the screening phytochemical, and all the other materials for the oocyst incubation that were necessary for the sporulation were obtained from the Pharmaceutical Sciences Research Laboratory, Faculty of Health Sciences, University of Lome. The culture media that was used was 2.5% potassium dichromate (K_2_Cr_2_O_7_).

For 1 L of PBS (Phosphate Buffer Saline) solution, 8 g of NaCl, 0.2 g of KCl, 1.44 g of Na_2_HPO_4_, and 0.24 g of KH_2_PO_4_ were put in 800 mL of distilled water. The mixture was adjusted with distilled water to obtain 1000 mL at a pH of 7.4. 

In total, 300 mM of acetate buffer solution, 3.10 g of sodium acetate trihydrate, and 16 mL of glacial acetic acid were adjusted to a pH of 3.6 and made up to a final volume of 1 L using distilled deionized H_2_O.

In total, 40 mM of a HCl (mr 36.46; SG = 1.18; and purity 35–38%) solution and 3.4 mL of concentrated HCl were made up to a final amount of 1 L using distilled deionized H_2_O. 

### 2.2. Plant Collection and Extract Preparation 

The whole leaves of *Combretum micranthum*, *Vernonia amygdalina*, *Azadirachta indica*, and *Carica papaya* seeds were collected from Badja, Ave district, while the *Combretum micranthum* leaves were collected from Notse. The roots of *Sarcocephalus latifolius* were obtained from the Gbossime market in Lome, Togo. The plant materials were authenticated at the Botany and Ecology Department of the University of Lome. After being cleaned, the roots, seeds, and leaves were dried at 20 °C under air conditioning, and an electric grinder was used to grind them into a powder. A hydro-ethanolic extract was prepared according to the method described by Bakoma et al. [12]. Briefly, 3 L of an ethanol/water solution (70:30 ratio) was soaked with 300 g of plant powder for 72 h at room temperature and was automatically stirred using an SM-30 CONTROL stirrer (Edmund Bühler GmbH, Bodelshausen, Germay). The mixtures were then filtrated three times with hydrophilic cotton and two times with Whatman filter paper, and the filtrate of each plant was evaporated at 45 °C using a BUCHI R-210 rotary evaporator (Marshall Scientific, Hampton, NH, USA).

### 2.3. Plant Extract Yields 

The yield of each plant was calculated for each extract using the following formula of Toah et al. [13] after the solvent evaporation and stored in bottles at 4 °C in the refrigerator till usage.

Yield = Mass of extract after evaporation (g)/mass of dried plant powder (g) × 100

### 2.4. Phytochemical Screening

The phytochemical analysis was conducted in the Pharmaceutical Sciences Research Laboratory, Faculty of Health Sciences, University of Lome, Togo. The tests for the presence or absence of phenols, tannins, saponins, flavonoids, alkaloids, reducing sugar, coumarins, triterpenes, and steroids are displayed in Table 1 in accordance with the standard assay protocol as stipulated by Karumi et al. [14].

### 2.5. Quantitative Phytochemical Analysis

#### 2.5.1. Determination of Total Polyphenol Content

The total polyphenol content of the extract was determined using the Folin–Ciocalteu method [15] with some modifications. A 1000 μL volume of the Folin–Ciocalteu solution diluted to a tenth was added to 200 μL of the extract, and the mixture was then incubated for 2 min at room temperature. Subsequently, 800 μL of 75 g·L^−1^ Sodium carbonate was added. After the combination was well mixed and allowed to sit at room temperature for 30 min, with its light source shielded, the absorbance reading was measured at 765 nm using a spectrophotometer. A calibration curve was generated using gallic acid standards at concentrations ranging from 0 to 100 μg·mL^−1^. Three replicates were performed for each extract. The total content was expressed as mg gallic acid equivalents (GAE)/g dry weight (DW).

#### 2.5.2. Determination of Total Flavonoids 

The method employed was as described by Bakoma et al. [16]. A 2 mL volume of 2% (*w*/*v*) AlCl3 in pure methanol was combined with an equal volume of the extract (1 mg/mL) in methanol. The absorbance was measured after 10 min in the dark at 415 nm using a spectrophotometer, with quercetin utilized as a reference substance. Three tests per extract were conducted, and the results were expressed in milligrams of quercetin equivalent (mg EqQ) per milligram of extract. The standard range (from 0 to 100 mg/L of quercetin) was carried out under the same conditions.

### 2.6. In Vitro Antioxidant Assays

#### 2.6.1. DPPH (2,2-Diphenyl-1-picrylhydrazyl) Free Radical Scavenging Activity

Using a stable free radical and the 2,2′-diphenQyl-1-picrylhydrazyl (DPPH) test, which was described by Bakoma et al. [16], the extracts’ ability to scavenge free radicals was evaluated. In order to assess each extract’s antioxidant activity (AAO), 100 µL of a methanol-dissolved extract containing 1 mg/mL of DPPH (0.004%) was added to 2 mL of a methanolic solution. To make sure the mixture was homogeneous, it was vortexed forcefully. A spectrophotometer was used to measure the absorbance at 517 nm after 30 min in the dark. For every sample, three separate tests were run. As a positive control, a solution of a standard antioxidant, quercetin (with concentrations ranging from 0 to 100 mg/mL), was prepared, and its absorbance was measured under the same conditions as the sample. The results obtained from the calibration curve equation were expressed in the milligram equivalent of quercetin per milligram of extract (mg EqQ/mg sample).

#### 2.6.2. Ferric Reducing Antioxidant Power (FRAP) Assay

The revised protocol of Benzie and Strain [17] was used for the ferric reducing antioxidant power (FRAP) test. The effectiveness of this technique depends on the sample’s ability to convert ferrous tripyridyltriazine (Fe(II)-TPTZ) into ferric tripyridyltriazine (Fe(III)-TPTZ) at a low pH. The formation of Fe(II)-TPTZ results in a strong blue color, which can be read at 593 nm. Specifically, 100 μL of the extracts were combined with 2 mL of freshly made FRAP solution, which contained 25 mL of 300 mM of acetate buffer at a pH of 3.6, 2.5 mL of 10 mM 2,4,6-tripyridyls-triazine (TPTZ) in 40 mM of HCl, and 2.5 mL of H_2_O for the standard FRAP, and 20 mM of ferric chloride {FeCl_3_·6H_2_O} solution for the sample FRAP. After 30 min in the dark, the absorbance was measured at 593 nm. Within a range of 0 to 100 μM of FeSO_4_·7H_2_O, the standard curve was linear. A comparison was made between the results and ascorbic acid in terms of the μM of Fe(II)/g dry plant material.

### 2.7. Eimeria Oocyst Isolation and Purification 

Fresh fecal samples from naturally infected broiler chickens were obtained from ninety-two poultry farms in the Maritime Region of Togo between July and September 2023. The oocysts were isolated from the fresh fecal matter using the procedure described by Flores et al. [18]. Phosphate-buffered saline (PBS) (at a pH of 7.4) was used to dilute the fecal samples five times, before they were homogenized with a vortex mixer, and filtered through a mesh screen. Afterwards, the filtrate was moved to a polypropylene centrifuge container and spun at 1000× *g* for ten minutes. The oocyst-containing sediment was re-suspended in PBS and centrifuged twice for ten minutes at 1000× *g* to wash it twice. For 72 h, sporulation was carried out in an aqueous solution of 2.5% (*w*/*v*) potassium dichromate [19]. Then, 2 mL of sediment was re-suspended with 2 mL of potassium dichromate solution (2.5%) in small Petri dishes. This was to provide enough moisture, as well as to kill the other microorganisms present in the samples that were competing for oxygen and nutrients with the oocysts. To induce sporulation, the samples were incubated for one to three days at 28 °C in an oven with aeration. The sporulation of oocysts was assessed with a 40× magnification microscopy, and an Olympus compound microscope equipped with an IX73 digital camera that was used to obtain pictures of both the sporulated and unsporulated oocysts.

The main morphological features, such as the size, shape, and morphological characteristic of the oocysts used, were described according to the key given by McDougald [20]. The morphological identification revealed the presence of *E. maxima* (54.17%), *E. brunetti* (33.33%), *E. tenella* (25%), *E. acervulina* (8.33%), *E. praecox* (8.33%), and *E. mitis* (4.17%).

### 2.8. Effect of Herbal Extracts on Oocyst Sporulation In Vitro 

**Figure 1:**
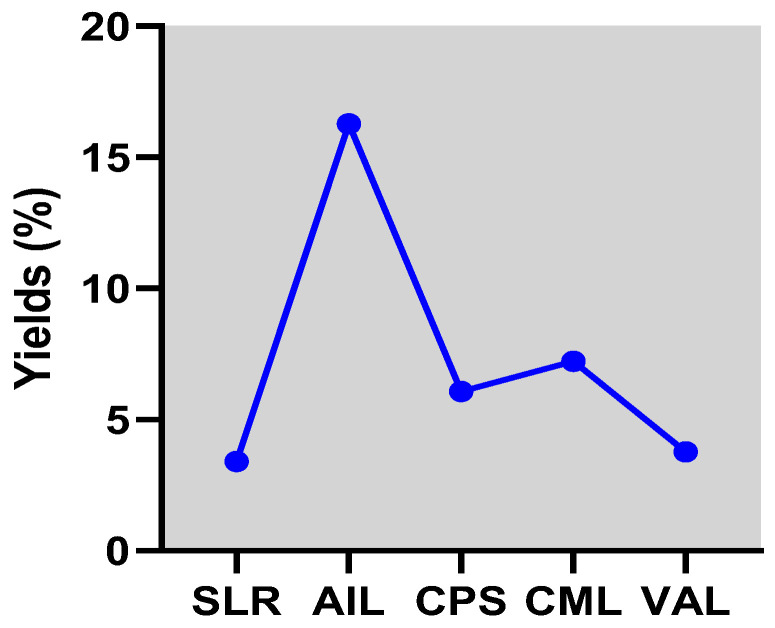
The yields of the hydro-ethanolic extracts from the medicinal plants used against coccidiosis in Togo. SLR, AIL, CPS, CML, and VAL represent the hydro-ethanolic extracts of *Sarcocephalus latifolius*, *Azadirachta indica*, *Carica papaya*, *Combretum micranthum*, and *Vernonia amygdalina*, respectively.

The impact of crude hydro-ethanol extracts of *Vernonia amygdalina* leaves (VALs), *Sarcocephalus latifolius* roots (SLRs), *Carica papaya* seeds (CPSs), *Azadirachta indica* leaves (AILs), and *Combretum micranthum* leaves (CMLs) on the sporulation time of *Eimeria* spp. oocysts with three replicates for each group was used to evaluate the in vitro anticoccidial activity. The experimental design is presented in Table 2. The experiment was performed following the method described by Baies et al. [21] in Petri dishes (60 × 15 mm) with a positive control containing 60 mg/L of Amprolium 20% and a negative control containing two milliliters of 2.5% potassium dichromate. Five different concentrations (6.25, 12.5, 25, 50, and 75 mg/mL) were made for each crude hydro-ethanolic extract from the selected plants. A total volume of 2 mL of each variant was placed in Petri dishes and 2 mL suspensions of freshly unsporulated oocysts of *Eimeria* spp. were dispensed. All the groups’ mix suspensions were semi-covered and incubated at 27–29 °C with a relative humidity of 65–75% [22] in a AVNTEC incubator, ETIN 0062. After 24, 48, and 72 h, one-milliliter suspensions from each Petri dish were transferred to test tubes; the oocysts were washed from the test solutions by mixing with PBS and then centrifuged at 200 g three times for five minutes. Using a light microscope (IX73, OLYMPUS, Tokyo, Japan) at a 40× magnification, a microscopic examination of each group was carried out. The sporulated oocysts with sporocysts, deformed walls, and inhibitory oocysts were examined with a light microscope fitted with a McMaster chamber (Figure 1). The percentages of sporocysts and inhibitory oocysts were calculated using the following formulae [23]:

$$Sporulation\left(\%\right)=\frac{oocysts\;number\;that\;has\;sporulated}{total\;number\;of\;oocysts}\times 100$$

Additionally, the methods outlined by Debbou-Iouknane et al. [24] were used to determine the lethal concentration (LC_50_) for each selected plant extract.

### 2.9. Statistical Analysis

For each of the 24 h, 48 h, and 72 h intervals, the mean and standard deviation of the mean were determined. Afterwards, the experimental groups were compared to each other and to the control groups using the ANOVA statistic and subsequently Duncan’s multiple range test. The Statistical Package for Social Science (SPSS version 22, Chicago, USA) was used. The percentage inhibition was analyzed by a two-way ANOVA using the General Linear Models procedure. The following model was used:

Y_ijk_ = μ + α_i_ + β_j_ + (αβ)_ij_ + ε_ijk_,

where Y_ijk_ represents an individual observation; μ is the experimental mean; α_i_ signifies the effect of the ith treatment; β_j_ indicates the effect of the jth time of incubation; (αβ)_ij_ is the effect of the treatment multiplied by the time of the incubation interaction; and ε_ijk_ signifies the random error.

All the graphs were made with the GraphPad software (Prism 8.3.0). The results were expressed as the mean ± SE. The probability values of less than 0.05 (*p* < 0.05) were considered significant.

## 3. Results

### 3.1. Extract Yield and Phytochemical Screening Assessment

This study analyzed the extract yields and phytochemical constituents of the most medicinal selected plants from the pharmacopeia used as antiparasitic herbal extracts in Togo poultry farms. The hydro-ethanolic extract yields (Figure 1) were as follows: *Azadirachta indica* had the highest yield at 16.27%, followed by *Combretum micranthum* (7.23%), *Carica papaya* (6.07%), *Vernonia amygdalina* (3.77%), and *Sarcocephalus latifolius* (3.41%). 

The bioactive compounds identified through the phytochemical screening analysis of the hydro-ethanolic plant extracts were as follows: phenolic compounds, tannins, saponins, flavonoids, alkaloids, reducing sugars, coumarins, quinones, triterpenes, and steroids. All these phytochemical elements were detected in the hydro-ethanolic extracts of *Sarcocephalus latifolius* and *Azadirachta indica*, except for quinones (Table 3).

### 3.2. Determination of Total Phenols and Flavonoid Content 

The total flavonoid and phenol concentrations presented in Table 4 were determined from the linear regression curves for phenols and flavonoids, respectively (y = 0.02910x + 0.1172; R^2^ = 0.9766 and y = 0.01527x + 0.05453; R^2^ = 0.9946) (Table 4). The total phenol levels obtained were 28.56 ± 0.27 mg EqAG/g for the *Azadirachta indica* leaf extract, 26.19 ± 0.19 mg EqAG/g for the *Combretum micranthum* leaves, 32.99 ± 0.36 mg EqAG/g for the *Carica papaya* seed extract, 56.11 ± 0.33 mg EqAG/g for the *Sarcocephalus latifolius* root extract, and 16.23 ± 1.17 mg EqAG/g for the *Vernonia amygdalina* leaves. The *Sarcocephalus latifolius* root presented the best phenol content compared to the other extracts (*p* = 0.001). The flavonoid levels obtained were 21.97 ± 0.97 mg EqQ/g for the *Azadirachta indica* leaf extract, 23.71 ± 0.38 mg EqQ/g for the *Combretum micranthum* leaf extract, 1.00 ± 0.08 mg EqQ/g for the *Carica papaya* seed extract, 36.65 ± 1.85 mg EqQ/g for the *Sarcocephalus latifolius* root extract, and 29.14 ± 1.79 mg EqQ/g for the *Vernonia amygdalina* leaves. The *Carica papaya* seed extract was the weakest in the selected plant extracts (*p* < 0.0001). 

### 3.3. Antioxidant Test Procedure 

#### 3.3.1. Free Radical Scavenging Activity of DPPH (2,2-Diphenyl-1-picrylhydrazyl)

The standard range was used to plot a linear regression line of the following equation: y = −0.01043x + 1.286; R^2^ = 0.9933. The antioxidant activity of the *Azadirachta indica* leaf extract was equivalent to 35.92 ± 0.39 mg of quercetin per gram of extract (mg Eq/g). Similarly, the antioxidant activity of the *Sarcocephalus latifolius* root and *Carica papaya* seed extracts were determined to be equivalent to 76.25 ± 0.53 and 69.77 ± 0.48 mg of quercetin per gram of sample, respectively. While the *Combretum micranthum* leaf and *Vernonia amygdalina* leaf extracts were the lowest being equivalent to 27.88 ± 0.41 and 23.68 ± 0.61 mg of quercetin per gram (Table 5).

#### 3.3.2. Ferric Reducing Antioxidant Power (FRAP) of Extracts

Table 5 shows the total antioxidant activity of the extracts from the *Azadirachta indica* leaves, *Combretum micranthum* leaves, *Carica papaya* seeds, *Sarcocephalus latifolius* roots, and *Vernonia amygdalina* leaves, which were, respectively, 26.97 ± 0.25, 23.71 ± 0.38, 86.21 ± 4.28, 57.53 ± 0.54, and 29.14 ± 1.79 μM Fe(II)/g. The ascorbic acid, which was the positive control, measured 97.37 ± 0.86 μM Fe(II)/g. 

### 3.4. In Vitro Oocysticidal Activities of Plant Extracts

**Figure 2:**
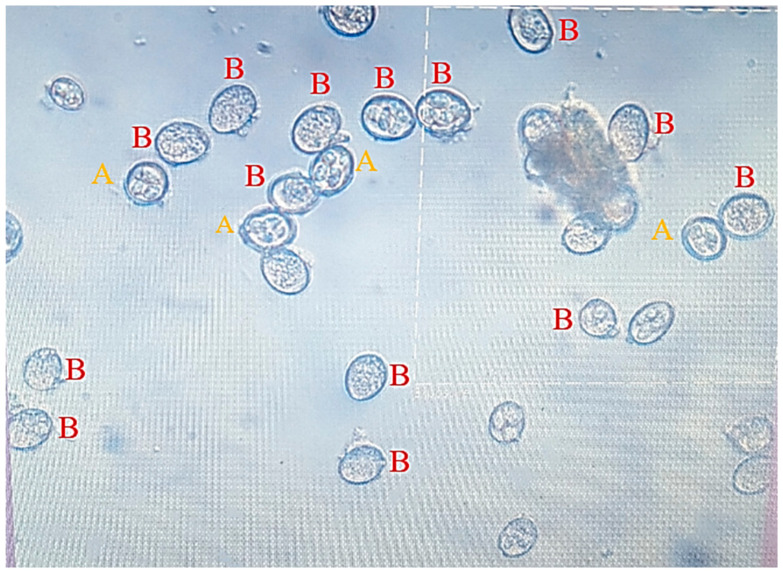
Microscopic observation of the changes that appeared on the oocysts when exposed to various treatments. **A** shows the sporulated oocysts; and **B** shows the unsporulated oocysts (400×).

Figure 2 displays a photomicrograph of both the sporulated and unsporulated oocysts. The sporulation of the oocysts was seen at several time points (24, 48, and 72 h) after the incubation. The sporocysts with sporozoites within were surrounded by two layers of outer and inner oocyst walls, which surrounded the oval-shaped sporulated *Eimeria* spp. after 72 h of incubation. 

**Figure 3:**
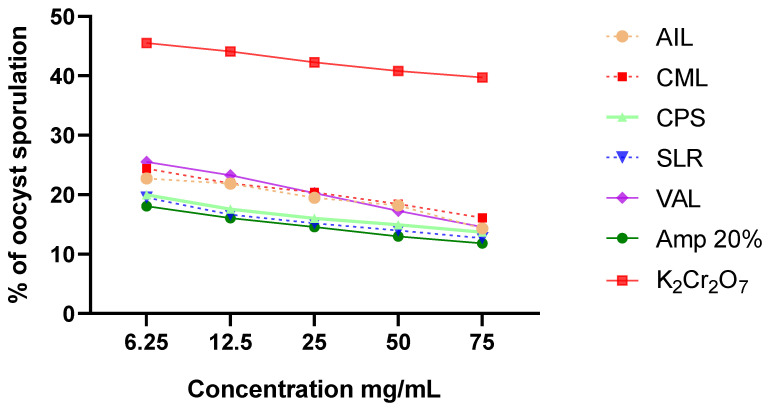
Effect of selected plant extracts on oocyst sporulation. The treatments were as follows: Amp 20%, Amprolium 20% (the positive control); K_2_Cr_2_O_7_, a 2.5% potassium dichromate solution (the negative control); and SLR, AIL, CPS, CML, and VAL, which represent hydro-ethanolic extracts of *Sarcocephalus latifolius*, *Azadirachta indica*, *Carica papaya*, *Combretum micranthum*, and *Vernonia amygdalina*, respectively.

The results demonstrated that all these potent antiparasitic plants utilized in Togo’s poultry farming significantly impacted the oocyst sporulation process of *Eimeria* spp. (*p* < 0.0001) compared to both control groups (the negative control and the reference therapy). After 72 h of incubation, the negative control (K_2_Cr_2_O_7_) exhibited a substantial oocyst sporulation rate of 89.72%, whereas the lowest sporulation rates were recorded for CPS 75 (26.47 ± 0.19%), SLR 75 (25.15 ± 0.38%), and Amp 20% (23.27 ± 1.19%) (Figure 3). For each of the examined plant species, the proportion of oocysts that completed the process of sporulation and the concentration of the plant extract showed an inverse proportional relationship (Figure 3).

**Figure 4:**
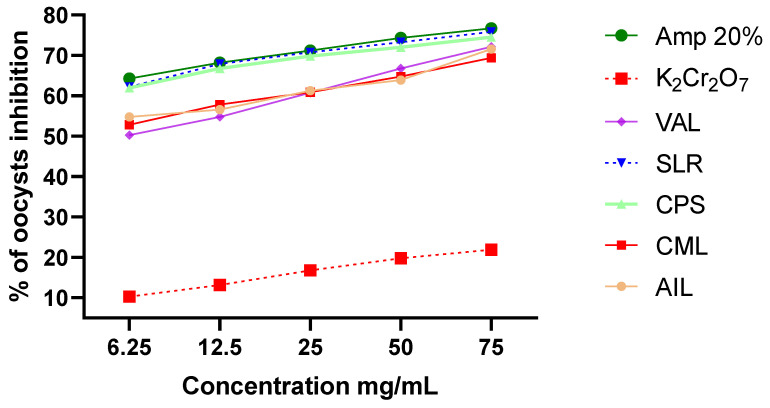
Effect of selected plant extracts on oocyst inhibition. The treatments were as follows: Amp 20%, Amprolium 20% (the positive control); K_2_Cr_2_O_7_, a 2.5% potassium dichromate solution (the negative control); and SLR, AIL, CPS, CML, and VAL, which represent hydro-ethanolic extracts of *Sarcocephalus latifolius*, *Azadirachta indica*, *Carica papaya*, *Combretum micranthum*, and *Vernonia amygdalina*, respectively.

After being incubated for 72 h with AILs, CPSs, SLRs, and Amprolium 20%, the *Eimeria* spp. showed lower sporulation levels than when 2.5% potassium dichromate was used as the negative control (Figure 4). The highest inhibition of sporulation was observed for AIL 75 (71.58 ± 0.24%), CPS 75 (74.53 ± 1.65%), SLR 75 (75.85 ± 1.21%), and Amprolium 20% (76.73 ± 0.34%).

**Figure 5:**
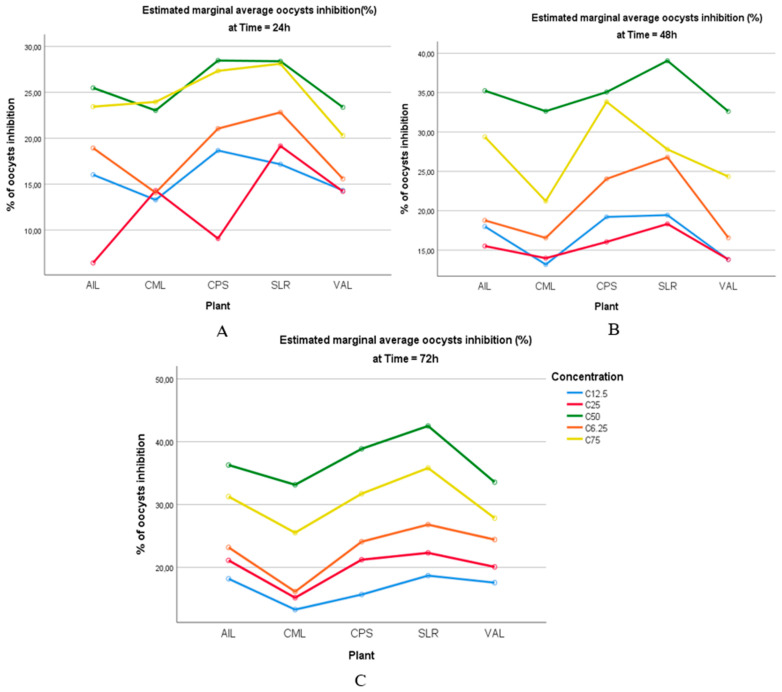
Effect of selected plant extracts on the inhibition of *Eimeria* oocysts at different concentrations at 24, 48, and 72 h in vitro. The treatments were as follows: Amp 20%, Amprolium 20% (the positive control); K_2_Cr_2_O_7_, a 2.5% potassium dichromate solution (the negative control); and SLR, AIL, CPS, CML, and VAL, which represent hydro-ethanolic extracts of *Sarcocephalus latifolius*, *Azadirachta indica*, *Carica papaya*, *Combretum micranthum*, and *Vernonia amygdalina*, respectively, at different concentrations and after 24 h (**A**), 48 h (**B**), and 72 h (**C**) of incubation.

Figure 5 depicts the comparative effects of the sporulation time, experimental groups, and plant extract concentrations on the inhibition percentage of *Eimeria* oocysts (%). The results indicate that the percentage of inhibition significantly increased with a prolonged incubation time (*p* < 0.05). Consequently, there were no significant differences in the sporulation inhibition rate among the Amprolium 20%, SLR, AIL, and CPS groups.

The minimum lethal concentration (LC_50_) of each selected plant extract is presented in Table 6. For the first two days post-incubation, the LC_50_ values were not calculated, as the extracts did not achieve 50% mortality of the oocysts. After being incubated for 72 h, the extract with the lowest lethal concentration was SLR, with an LC_50_ of 21.42 mg/mL, followed in order by CPS, AIL, VAL, and CML (as shown in Table 5).

## 4. Discussion

The prevention and control of coccidiosis represents a significant challenge for the poultry industry, due to the resistance of oocysts to physical and chemical treatments as a result of the proteinaceous layers of the oocyst walls [8]. Consequently, various botanicals and their products have shown promising activities in inhibiting oocyst sporulation, as demonstrated by several in vitro studies [25,26]. This research aimed to compare the bioactive components, antioxidant activities, and sporulation inhibition effects of SLRs, AILs, CPSs, CMLs, and VALs, traditionally used in Togo’s poultry farms as antiparasitic herbal extracts. The results showed variations in the yields of the hydro-ethanolic extracts of these plants, with SLRs having the lowest yield at 3.41%, and in the presence or absence of bioactive components in the plants. These variances could be attributed to the type of solvent used, the method of extraction, or the local climate conditions where the plant samples were collected from [27]. These results are similar to those observed by Yinusa et al. [28], who reported yields of 3.99% for the ethanol extracts of *Sarcocephalus latifolius.* The absence of tannins, alkaloids, and coumarins in the CPSs, CMLs, and VALs hydro-ethanolic extracts, compared to the SLR extract, demonstrates that a high yield does not necessarily mean the presence of bioactive components [29]. In order to investigate a potential relationship between the activity and the polyphenolic content, we also quantified the amounts of phenols and flavonoids in the most effective extracts. The results showed that the hydro-ethanolic extracts from the *Azadirachta* indica leaves, *Sarcocephalus latifolius* roots, and *Carica papaya* seeds contained the highest levels of phenolic compounds. This confirms the importance of these secondary metabolites for the control of coccidiosis, as evidenced by the significant reduction in oocyst counts reported by Murshed et al. and Dakpogan et al. [30,31] and the in vitro oocyst inhibitory potential described by Arlette et al. [32]. The ability of the selected plants to scavenge free radicals was evaluated. For both the DPPH and FRAP assays, the hydro-ethanolic extract of *Sarcocephalus latifolius* roots exhibited the strongest antioxidant capacity, followed by the *Carica papaya* seeds and *Azadirachta indica* extract. This free radical scavenging capacity of the plants may be primarily attributed to the high levels of phenolic compounds present in them [33] and to their polar nature, which can contribute to enhancing their free radical reduction potential [34]. Several authors have reported that the reducing power of the phenolic compound may serve as a predictor of their possible antioxidant properties [35]. The anticoccidial properties of these plants were mostly assessed through inhibition of the *Eimeria* oocyst sporulation in this study. A study showed a potential higher inhibition of oocyst sporulation when the anticoccidial capacity of the methanolic extract of *Annona reticulata* leaves was tested in vitro at 250, 125, 62.5, and 31.25 mg/mL for 72 h [8]. Likewise, in a similar study, the anticoccidial potential of *Pinus radiata* bark extract showed a significant decrease in the viability of sporulated oocysts at 1000 μg after 48 h in vitro [19]. Among the plant extracts, the SLRs, CPSs, AILs and the positive control presented the best levels of sporulation inhibition of the *Eimeria* oocysts compared to the other treatments. The high inhibition percentage of the SLR 75 (75.85%), CPS 75 (74.53), and AIL 75 (71.58%) extracts could be associated with the variations in the concentration of their various phytochemical compounds. The bactericidal nature of K_2_Cr_2_O_7_ may be the reason for its incapacity to prevent sporulation [36]. This study’s findings are consistent with those of Abbas et al. and El-Khtam [1,37], who documented the in vitro anticoccidial action of a *Trachsypermum ammi* extract on the oocysts of four different kinds of hens belonging to the Eimeria genus and the in vitro efficacy of garlic (about 80%) on the oocyst sporulation of mixed *Eimeria* species isolated from naturally infected chickens. The anticoccidial ability of pine bark (*Pious radiata*) extracts was observed to considerably reduce the sporulation of the oocysts of *E. tenella*, *E. maxima*, and *E. acervulina*, according to similar in vitro results published by Molan et al. [19]. In this current study, bioactive components such as phenols, tannins, saponins, flavonoids, alkaloids, reducing sugar, coumarins, triterpenes, and steroids were found in the plant extracts using a phytochemical screening analysis. The decrease in oocyst sporulation is a sign that the tannins and alkaloids contained in the plant extracts can impede the life cycle of coccidia. The low rates of sporulation inhibition observed may be explained by the absence of tannins and alkaloids in the CML and VAL extracts. Tanning agents work by penetrating the oocyst wall and subsequently inducing cytoplasmic damage by inhibiting the endogenous enzymes that are responsible for the sporulation process [19]. In chicken diets, several herbal extracts are frequently used to boost growth rates and animal health, particularly in cases where health issues are prevalent. Plant extracts have been shown in numerous studies to enhance the feed conversion ratio, weight gain, and feed intake in chickens [38,39]. It might be possible to use the anticoccidial properties of *Azadirachta indica* leaves, *Carica papaya* seeds, and *Sarcocephalus latifolius* roots to reduce coccidiosis in poultry farms. This in vitro study concludes that all of the selected hydro-ethanolic plant extracts exhibited a significant anticoccidial effect. Furthermore, this anticoccidial activity was directly proportional to the concentration of the plant extract used.

## 5. Conclusions

In summary, the findings of this study demonstrate the efficacy of herbal remedies, including *Sarcocephalus latifolius* roots, *Azadirachta indica* leaves, *Carica papaya* seeds, *Combretum micranthum* leaves, and *Vernonia amygdalina* leaves, in inhibiting *Eimeria* spp. sporulation in vitro. The results indicate that the extracts from *Sarcocephalus latifolius* roots, *Azadirachta indica* leaves, and *Carica papaya* seeds exhibit potent anti-*Eimeria* effects, which is attributed to their strong sporulation inhibition and anti-sporozoite activities, likely mediated by their flavonoids, tannins, and alkaloids and their high capacity to trap free radicals (their antioxidant capacity). These selected plant extracts could serve as alternative options for developing natural bioactive agents to combat coccidiosis, especially when conventional drugs like Amprolium are costly. Nevertheless, further investigations and in vivo trials are warranted to elucidate the mechanisms of action of these active compounds and assess the potential toxicity of these plant extracts.

## Figures and Tables

**Table 1:** Hydro-ethanolic plant extracts: a qualitative phytochemical screening method.

Phytoconstituents	Test/Reagents	Precipitation
Total phenols	Ferric chloride test	Blue precipitate
Tannins	Stiasny test	Blue for galli and green for catecholic
Saponin	Frothing test	Persistent foam
Flavonoids	Shinoda test	Red color
Alkaloids	Dragendorff’s test	Orange precipitate
Reducing sugar	Fehling’s solution	Brick red precipitate
Coumarins	Ammonium hydroxide	Fluorescent color at 360 nm
Quinones		
Triterpenes	Acetic anhydride, chloroform, and sulphuric acid	Red violet color
Steroids		Brick red ring

**Table 2:** Experimental design.

Groups	Concentrations (mg/mL)	Abbreviations
Hydro-ethanolic plant extracts	6.25	AIL6.25, CML6.25, CPS6.25, SLR6.25, CV6.25
	12.5	AIL12.5, CML12.5, CPS12.5, SLR12.5, CV12.5
	25	AIL25, CML25, CPS25, SLR25, CV25
	50	AIL50, CML50, CPS50, SLR50, CV50
	75	AIL75, CML75, CPS75, SLR75, CV75
Amprolium 20%	0.06	Amp25%
Potassium dichromate	2.5	K_2_Cr_2_O_7_

**Table 3:** Qualitative phytochemical screening of the plant extracts.

Screening Phytochemical	*Sarcocephalus latifolius*	*Carica papaya*	*Combretum micranthum*	*Vernonia amygdalina*	*Azadirachta indica*
	Rubiaceae	Caricaceae	Combretaceae	Asteraceae	Meliaceae
Total phenols	+	+	+	+	+
Tannins	+		+	-	+
Saponin	+	+	+	+	+
Flavonoids	+	+	+	+	+
Alkaloids	+	-	-	+	+
Reducing sugar	+	+	+	+	+
Coumarins	+	-	-	+	+
Quinones	-	-	-	-	-
Triterpenes	+	+	+	+	+
Steroids	+	+	+	+	+

**Table 4:** Total polyphenol and flavonoid contents of the hydro-ethanolic extracts (mean ± SE).

Plant Extracts	Total Phenolic and Flavonoid	
	Flavonoid (mg EQ/g)	Phenols (mg EAG/g)
*Azadirachta indica* leaves	21.97 ± 0.97 ^b^	28.56 ± 0.27 ^b^
*Combretum micranthum* leaves	19.33 ± 0.05 ^b^	26.19 ± 0.19 ^b^
*Carica papaya* seeds	1.00 ± 0.08 ^c^	32.99 ± 0.36 ^b^
*Sarcocephalus latifolius* roots	36.65 ± 1.85 ^a^	56.11 ± 0.33 ^a^
*Vernonia amygdalina* leaves	16.23 ± 1.17 ^b^	19.47 ± 0.28 ^b^
*p*-value	0.0001	0.001

SE: Standard Error, (values in the same column that not share the same superscript letters are significantly different, *p* < 0.05).

**Table 5:** Antioxidant activity assay (mean ± SE).

Plant Extracts	Antioxidant Activity	
	DPPH (mg EQ/g)	FRAP (μM Fe(II)/g)
*Azadirachta indica* leaves	35.92 ± 0.39 ^b^	26.04 ± 0.25 ^c^
*Combretum micranthum* leaves	27.88 ± 0.41 ^c^	23.71 ± 0.38 ^c^
*Carica papaya* seeds	69.77 ± 0.48 ^a^	57.53 ± 0.54 ^b^
*Sarcocephalus latifolius* roots	76.25 ± 0.53 ^a^	86.21 ± 4.28 ^a^
*Vernonia amygdalina* leaves	23.68 ± 0.61 ^c^	29.14 ± 1.79 ^c^
*p*-value	<0.0001	<0.0001

SE: Standard Error, (values in the same column that not share the same superscript letters are significantly different, *p* < 0.05).

**Table 6:** The minimum lethal concentration (LC_50_) of the selected plant extracts after 72 h of incubation.

Selected Plants	Lethal Concentration (LC_50_) (mg/mL)
*Azadirachta indica* leaves	26.35
*Combretum micranthum* leaves	31.24
*Carica papaya* seeds	24.56
*Sarcocephalus latifolius* roots	21.42
*Vernonia amygdalina* leaves	28.12

## Data Availability

All data contained within the article.
